# New York Dental Recorder

**Published:** 1854-04

**Authors:** 


*New York Dental Recorder.*—Dr. C. C. Allen, formerly and for many years the sole editor of the Dental Recorder, and during the last year the senior editor, has wholly withdrawn from the publication, confiding its future management to Dr. A. Hill, of Norwalk, Ct., who had previously for a short time, been associated with him as junior editor. We are sorry to lose so good a writer and so courteous and gentlemanly a man from our little corps editorial as Dr. A., but we at the same time cannot but congratulate him on being relieved from the cares and vexations necessarily connected with the management of a publication of this sort. Having experienced them ourselves, we know what they are. But while the readers of the Recorder, with us, regret the loss of the former able senior editor they must at the same time be gratified that it is still under the management and control of a gentleman every way qualified to conduct the publication. Dr. Hill, the present editor, has been known to the dental profession for several years as a ready writer and as a man of science. We extend to him, therefore, most cordially, the right hand of fellowship, and welcome him to all the pleasures, duties and responsibilities of full editorship, wishing him abundant encouragement and success in his arduous, and to some extent, new undertaking.

